# Effects of IAA, IBA, NAA, and GA3 on Rooting and Morphological Features of *Melissa officinalis* L. Stem Cuttings

**DOI:** 10.1155/2013/909507

**Published:** 2013-05-30

**Authors:** Hakan Sevik, Kerim Guney

**Affiliations:** ^1^Landscape Architecture Department, Faculty of Engineering and Architecture, Kastamonu University, 37150 Kastamonu, Turkey; ^2^Forest Engineering Department, Forestry Faculty, Kastamonu University, 37150 Kastamonu, Turkey

## Abstract

This study analyzed the potential of producing *Melissa officinalis* L. using stem cuttings. Four different hormones (IAA, IBA, NAA, and GA3) were applied to the cuttings, with and without buds, in two doses (1000 mg/L and 5000 mg/L), and after 60 days, 10 morphological characteristics of newly generated plants were detected, and a statistical analysis was carried out. The results of the study show that the cuttings with at least one bud must be used in order to produce *M. officinalis* using stem cuttings. Even though the auxin group hormones (IAA, IBA, and NAA) do not have an apparent effect on rooting percentage, these hormones were detected to affect the morphological characteristics of the newly generated plants, especially root generation. GA3 application has a considerable effect on stem height.

## 1. Introduction

Lemon balm (*Melissa officinalis *L.) belongs to the family Lamiaceae and grows widely in central and southern Europe and in Asia minor [1]. It is also found in tropical countries (Brazil) where it is popularly known as erva-cidreira and *Melissa* [2]. It is an aromatic (lemony) perennial herb, up to about 1 m high. Parts mostly used are dried leaves, often having flowering tops [3].

Green lemon aromatized leaves of this plant are used as fresh leaves, as well as in their dried form in salads, sauces, soups, with vegetables and meat, and in desserts. This plant is used in brewing certain alcoholic drinks and liquors and is consumed as an herbal tea. It is also used as an ornamental plant and as a border plant, particularly in gardens [4].

*M. officinalis *has been used in a variety of practical applications in medical science. The leaves contain volatile oils [5]. The leaf also contains polyphenolic compounds: caffeic acid derivatives in large proportions, such as rosmarinic acid, trimeric compounds, and some flavonoids [6].

*M. officinalis *can modulate a number of behavioral measures, with indications including administration as a mild sedative, in disturbed sleep, and in the attenuation of the symptoms of nervous disorders, including the reduction of excitability, anxiety, and stress [7]. *M. officinalis *extracts can attenuate the subjective effects of laboratory-induced stress [8]. It can be a useful herbal medicine for the treatment of gastrointestinal spasms [9]. *M. officinalis *L. has an antispasmodic and antimeteoric activity, as previously shown in animals and humans [10]. Schnitzler et al. [11] reported that *M. officinalis* oil might be suitable for topical treatment of herpetic infections. It has acetylcholine receptor activity in the central nervous system, with both nicotinic and muscarinic binding properties [12].

*M. officinalis *is predominantly sold in combination with other herbs, with, as an illustration, 49 products containing lemon balm in the German pharmaceutical industry's current “Rote Liste” (2001) drug catalogue [13]. From its Moorish introduction into Spain in the seventh century, its cultivation and use spread throughout Europe by the middle ages [13]. It is cultivated throughout the world because of its culinary properties [1].

Due to its economic importance, this plant is produced in large fields in several European countries such as France, Germany, Bulgaria, and Romania as well as in North America. Even though the lemon balm plant naturally spreads on the flora of our country, its agriculture level is not satisfactory, and occasionally it is collected from nature and exported [14].

A significant amount of the lemon balm that is widely used in Turkey is collected from the natural flora. This includes cultivation of lemon balm, which has a very high economic importance and will prevent the excessive and senseless destruction of natural flora, at least to a certain extent. In this study, the potential of producing *M. officinalis *L. using stem cuttings was analyzed. For this purpose, four different hormones were applied to the stem cuttings in two doses, and the effect of nine applications, together with the control group, on 10 morphological characteristics was analyzed. 

## 2. Materials and Methods

*M. officinalis *cuttings used in this research were collected from the Daday district of the province of Kastamonu in Turkey. The cuttings were collected on September 8, moisturized, and stored in germination turf. The applications on the cuttings brought to the laboratory were carried out on September 9, as explained hereinafter.

- Two-thirds of the 3 × 3 × 15 cm polyethylene tubes were filled with Klasmann germination turf in the laboratory.
- The cuttings were cut to 2.5 cm long pieces using sterile lancets and grouped as cuttings with and without buds.
- Solutions of four different hormones in two different doses (1000 mg/L and 5000 mg/L) were applied to the cuttings, respectively, and nine application groups were created, consisting of eight hormone application groups and a control group. The applications were carried out by imbruing the cuttings in hormone for 4 to 5 minutes. This application was composed of 3 replications and 15 cuttings in each replicate (15 cuttings with buds, and 15 cuttings without buds). Five cuttings were placed in each tube without any contact, covered with approximately 2 cm germination turf, and moisturized immediately.
- These tubes were placed in boxes with holes at room temperature (20–25°C), did not receive direct sun light, and were watered two times a day for 60 days. As the turf reached the saturation point, no water accumulation was generated, as the tubes and the boxes the tubes were placed in contained holes, and the surplus water was released.
- Measurements were carried out on November 8, which is the 60th day of the procedure. The turf in the tubes was poured on a laboratory bench, the roots were cautiously cleaned, and the number of the roots was defined. The average length of the roots was measured using a digital microcompass. After this procedure, stems and leaves were measured. All of these measurements were carried out using a digital microcompass with 0.01 mm precision, and the results were entered in a table. Ten morphological characteristics for each cutting were defined at the end of the study, including rooting percentage (RP), stem length (SL), stem length without branch (SLB), diameter (SD), number of leaves (LN), length of leaves (LL), size of leaf blade (LS), width of leaf blade (LW), number of roots (RN), and length of root (RL).

Variance analysis was applied on the data, using the SPSS 17.0 package program. The Duncan test was applied for the characteristics with at least 95% level of statistical reliance, and as a result, homogenous groups were acquired and interpreted. 

## 3. Results

At the end of the study, no new stem was formed in the stem cuttings without buds. This result implies that the cuttings with buds must be used while producing *Melisa officinalis *with stem cuttings. Hormone applications in different doses affect the germination percentages, as well as the characteristics of the germinated individuals, at different levels. The data acquired with the results of the study, the results of variance analysis applied on these data, and the Duncan test are given in Table 1. 

When the values stated in the table are analyzed, no germination is observed in the IAA hormone at a 5000 mg/L dose. The highest germination percentage values were acquired with 1000 mg/L IAA and 1000 mg/L GA3 hormone applications. The values acquired as a result of these applications are higher than the germination values acquired in the control group; however, according to the results of the Duncan test, these values are in the same homogenous group with the control group. According to the results of the Duncan test, the first homogenous group is composed of the application in which no germination was observed; 1000 mg/L IAA and 1000 mg/L GA3 applications together with the control group are included only in the second homogenous group, and the other applications were included in both homogenous groups. 

According to the values stated in the table, significant differences with a 95% statistical reliance level arose between the applications; however, the statistical reliance level of the differences arose in accordance with the other characteristics was 99.9%. This result indicates that even though the hormone applications did not reveal the expected effect on germination percentage, they have a considerable effect on other characteristics. 

When the values stated in the table are analyzed, it is observed that the longest seedlings with 81.72 stem length without branches and 96.75 total stem length were produced with the 5000 mg/L GA3 application. The seedlings produced in the control group have 55.57 total length and 30.12 stem length without branches. In this case, the length of the 5000 mg/L GA3 seedlings is 2.71 times greater than the control group in terms of stem length without branches and 74% greater in total length. Similarly, the length of 1000 mg/L GA3 applied seedlings is 87% greater than that of the seedlings in the control group, in terms of stem length without branches, and 38% greater in total length. However, the seedlings produced as a result of other applications are either shorter than the control group or included in the same homogenous groups with the control group according to the result of the Duncan test. 

The highest values of seedling diameter and number of leaves were acquired with the 5000 mg/L IBA application; however, one of the greatest values of the number of leaves was obtained in the control group. As a result, it can be concluded that the hormone applications do not have a positive effect, especially on the number of leaves.

When the effect of hormone applications on the leaf size is analyzed, it is observed that the IBA applications have a high effect on the leaf size and width. It was observed that the leaves exposed to the 1000 mg/L IBA application are 55% longer and 45% wider compared to the control group. The leaves exposed to the 5000 mg/L IBA application are 31% longer and 44% wider than the control group. It is observed that the 5000 mg/L IBA application is highly effective in terms of total leaf size. The leaves produced in the control group are 18.84 mm, while this figure is 27.38 mm in the 5000 mg/L IBA application and 23.1 mm in the 5000 mg/L GA3 application, which is highly effective in terms of increase in length of the seedlings. 

Even though the stem length is an important indicator of the sapling quality, root/stem ratio is very important for a healthy sapling. The saplings that can generate hairy roots are generally accepted to be healthier, and the saplings that can generate taproot in a short span of time reach ground water more easily in the natural environment, and thus, their chance of survival increases. 

Accordingly, root generation is one of the most important sapling quality indicators. According to the results of the study, the saplings in the control group developed 2.67 roots with an average length of 10.32, while the saplings receiving the 1000 mg/L IBA application developed 4 roots with an average length of 54.02. The saplings that received the 5000 mg/L IBA application had 5.5 roots with an average length of 21.35. The saplings that received the 5000 mg/L GA3 application developed 4 roots with an average length of 13.81. 

The results of the study show that the hormone applications have a great effect on root development. The fact that only the number of roots of the seedlings developed with the 1000 mg/L GA3 application is lower than that of the control group, while all of the applications developed longer roots compared to the control group, and the roots of the saplings developed with the 1000 mg/L IBA application are more than five times longer than those of the control group indicates that the hormone applications have a great effect on root development. 

## 4. Discussion

The results of the study demonstrate that cuttings with at least one bud must be used in order to produce *M. officinalis *using stem cuttings. No rooting developed in the stem cuttings without buds. 

The results of the applications show that the auxin group of hormones (IAA, IBA, and NAA), the subject of this study, do not have an apparent effect on rooting rate but do have an effect on the morphological characteristics of newly generated plants. Root development in particular reached significantly different values in the plants that received the auxin group of hormones. 

The process of adventitious root formation is influenced by a number of internal and external factors. Among the internal factors, the most important role is ascribed to phytohormones, especially the auxins. It is generally accepted that auxins have a certain role in the rooting initiation [15]. Auxins control growth and development in plants, including lateral root initiation and root gravity response. Many studies have shown that exogenous application of auxins results in increased initiation of lateral roots and that lateral root development is highly dependent on auxin and auxin transport [16]. 

The effects of auxin group of hormones on rooting and plant development have been discussed in several studies. Alvarez et al. [17] analyzed the effectiveness of IAA and IBA in *Malus pumila*; Štefančič et al. [15] studied the effectiveness of IAA and IBA in *Prunus* spp. as well as IBA and NAA in *Pseudotsuga menziesii*; Hossain et al. [18] analyzed the effectiveness of IBA in *Swietenia macrophylla *and *Chukrasia velutina*; Hussain and Khan [19] analyzed the effectiveness of IAA and IBA in *Rosa *species; Ozel et al. [20] analyzed the effectiveness of IAA and NAA in *Centaurea tchihatcheffii*; Chhun et al. [16] researched the effectiveness of IAA, IBA, and NAA in *Oryza sativa*; De Klerk et al. [21] analyzed the effectiveness of IAA, IBA, and NAA in *Malus*; Martin [22] studied the effectiveness of IBA in *Holostemma ada-kodien*; Nordström et al. [23] researched the effectiveness of IAA and IBA in *Pisum sativum*; Tchoundjeu et al. [24] analyzed the effectiveness of IBA in *Prunus Africana*; Swamy et al. [25] studied the effectiveness of IBA and NAA in both *Robinia pseudoacacia* and *Grewia optiva*. The studies show that, in general, the auxin group of hormones has an effect on rooting. This result is in conformity with the results of this study. 

Gibberellins are in the third place with a 17% share among the most commonly used herbal hormones within the natural plant growth regulators. Commercially the most common gibberellin is GA3, and it is used to increase the length of the plant or to enhance plant yield [26]. The results of the study reveal that the length of seedlings receiving the 5000 mg/L GA3 application is 2.71 times greater than that of the control group in terms of stem length without branches and 74% greater in total length. This result is also in compliance with the literature results. 

Effect of GA3 on rooting was also analyzed in several studies. The efficiency of GA3 on *Prunus avium *L. and *Prunus mahaleb *was analyzed by Hepaksoy [27], by Aygün and Dumanoğlu [28] on *Cydonia oblonga*, by Coşge et al. [29] on *Capparis ovata *and *Capparis spinosa*, and by Selby et al. [30] on *Picea sitchensis*. However, no apparent GA3 efficiency on rooting was detected in several species. 

## Figures and Tables

**Table 1:** Mean values of characters, results of variance analysis, and the Duncan test.

Hormone	Dose	RP	SL	SLB	SD	LN	LL	LS	LW	RN	RL
IAA	1000	44,0^b^	41,55^a^	16,56^a^	1,03^b^	5,00^b^	21,55^de^	12,27^abc^	16,45^cd^	3,0^bc^	10,74^ab^
IAA	5000	0^a^									
IBA	1000	17,2^ab^	60,71^bc^	37,46^bc^	1,07^b^	6,00^b^	10,02^a^	20,49^e^	19,27^d^	4,0^d^	54,02^e^
IBA	5000	8,0^ab^	52,83^ab^	32,71^bc^	1,18^c^	6,00^b^	27,38^f^	17,22^de^	19,23^d^	5,5^e^	21,35^cd^
NAA	1000	14,6^ab^	60,93^bc^	41,40^c^	0,79^a^	6,00^b^	16,81^bcd^	13,39^bc^	14,02^bc^	3,0^bc^	15,92^abcd^
NAA	5000	8,0^ab^	62,52^ab^	51,36^cd^	0,85^a^	3,44^a^	15,43^bc^	10,83^a^	10,66^a^	3,0^cd^	22,49^d^
GA3	1000	42,6^b^	76,42^c^	56,47^d^	0,83^a^	3,75^a^	14,49^ab^	10,86^ab^	10,30^ab^	2,0^a^	18,26^bcd^
GA3	5000	14,6^ab^	96,75^d^	81,72^e^	0,89^a^	5,50^b^	23,10^ef^	14,64^cd^	12,65^abc^	4,0^d^	13,81^abc^
Control	0	41,2^b^	55,57^ab^	30,12^ab^	0,93^b^	6,00^b^	18,84^cde^	13,18^bc^	13,31^abc^	2,67^ab^	10,32^a^
*F* values		1,993	12,09	25,617	17,471	9,316	11,898	9,865	8,77	35,778	23,273
Significant		0,044	0,000	0,000	0,000	0,000	0,000	0,000	0,000	0,000	0,000

## References

[1] Dastmalchia K, Damien Dormana HJ, Oinonena PP, Darwisd Y, Laaksoa I, Hiltunena R (2008). Chemical composition and in vitro antioxidative activity of a lemon balm (*Melissa officinalis* L.) extract. *Food Science and Technology*.

[2] de Sousa AC, Alviano DS, Blank AF, Barreto Alves P, Alviano CS, Gattass CR (2004). *Melissa officinalis* L. essential oil: antitumoral and antioxidant activities. *Journal of Pharmacy and Pharmacology*.

[3] Herodez S, Hadolin M, Skergeta M, Knez Z (2003). Solvent extraction study of antioxidants from Balm (*Melissa officinalis* L.) leaves. *Food Chemistry*.

[4] Kızıl S (2009). The effect of different harvest stages on some agronomical characteristics of Lemon balm (*Melissa officinalis* L.). *Tarim Bilimleri Dergisi*.

[5] Allahverdiyev A, Duran N, Ozguven M, Koltas S (2004). Antiviral activity of the volatile oils of *Melissa officinalis* L. against Herpes simplex virus type-2. *Phytomedicine*.

[6] Carnat AP, Carnat A, Fraisse D, Lamaison JL (1998). The aromatic and polyphenolic composition of lemon balm (*Melissa officinalis* L. subsp. *officinalis*) tea. *Pharmaceutica Acta Helvetiae*.

[7] Kennedy DO, Wake G, Savelev S (2003). Modulation of mood and cognitive performance following acute administration of single doses of *Melissa officinalis* (Lemon balm) with human CNS nicotinic and muscarinic receptor-binding properties. *Neuropsychopharmacology*.

[8] Kennedy DO, Little W, Scholey AB (2004). Attenuation of laboratory-induced stress in humans after acute administration of *Melissa officinalis* (lemon balm). *Psychosomatic Medicine*.

[9] Sadraei H, Ghannadi A, Malekshahi K (2003). Relaxant effect of essential oil of *Melissa officinalis* and citral on rat ileum contractions. *Fitoterapia*.

[10] Savino F, Cresi F, Castagno E, Silvestro L, Oggero R (2005). A randomized double-blind placebo-controlled trial of a standardized extract of *Matricariae recutita*, *Foeniculum vulgare* and *Melissa officinalis* (ColiMil) in the treatment of breastfed colicky infants. *Phytotherapy Research*.

[11] Schnitzler P, Schuhmacher A, Astani A, Reichling J (2008). *Melissa officinalis* oil affects infectivity of enveloped herpesviruses. *Phytomedicine*.

[12] Akhondzadeh S, Noroozian M, Mohammadi M, Ohadinia S, Jamshidi AH, Khani M (2003). *Melissa officinalis* extract in the treatment of patients with mild to moderate Alzheimer’s disease: a double blind, randomised, placebo controlled trial. *Journal of Neurology Neurosurgery and Psychiatry*.

[13] Kennedy DO, Scholey AB, Tildesley NTJ, Perry EK, Wesnes KA (2002). Modulation of mood and cognitive performance following acute administration of *Melissa officinalis* (lemon balm). *Pharmacology Biochemistry and Behavior*.

[14] Kaçar O, Göksu E, Azkan N (2010). The effect of different plant densities on some agronomic characteristics at lemon balm (*Melissa officinalis* L.) Cultivation. *Journal of Agricultural Faculty of Uludag University*.

[15] Štefančič M, Štampar F, Osterc G (2005). Influence of IAA and IBA on root development and quality of Prunus “GiSelA 5” leafy cuttings. *HortScience*.

[16] Chhun T, Taketa S, Tsurumi S, Ichii M (2003). The effects of auxin on lateral root initiation and root gravitropism in a lateral rootless mutant *Lrt1* of rice (*Oryza sativa* L.). *Plant Growth Regulation*.

[17] Alvarez R, Nissen S, Sutter EG (1989). Relationship between Indole-3-Acetic Acid Levels in Apple (*Malus pumila* Mill) rootstocks cultured in vitro and adventitious root formation in the presence of lndole-3-Butyric Acid. *Journal of Plant Growth Regulation*.

[18] Hossain MA, Islam MA, Hossain MM (2004). Rooting ability of cuttings of *Swietenia macrophylla* King and *Chukrasia velutina* wight et arn as influenced by exogenous hormone. *International Journal of Agriculture & Biology*.

[19] Hussain A, Khan AM (2004). Effect of growth regulators on stem cutting of *Rosa bourboniana* and *Rosa gruss-an-teplitz*. *International Journal of Agriculture & Biology*.

[20] Ozel CA, Khawar KM, Mirici S, Arslan O, Ozcan S (2006). Induction of *Ex Vitro* adventitious roots on soft wood cuttings of *Centaurea tchihatcheffii tchihatcheffii* Fisch et. Mey usingIndole 3-butyric acid and *α*-naphthalene acetic acid. *International Journal of Agriculture & Biology*.

[21] De Klerk GJ, Ter Brugge J, Marinova S (1997). Effectiveness of indoleacetic acid, indolebutyric acid and naphthaleneacetic acid during adventitious root formation in vitro in Malus ‘Jork 9’. *Plant Cell, Tissue and Organ Culture*.

[22] Martin KP (2002). Rapid propagation of Holostemma ada-kodien Sehult., a rare medicinal plant, through axillary bud multiplication and indirect organogenesis. *Plant Cell Reports*.

[23] Nordström AC, Jacobs FA, Efiasson L (1991). Effect of exogenous indole-3-acetic acid and indole-3-butyric acid on internal levels of the respective auxins and their conjugation with aspartic acid during adventitious root formation in pea cuttings. *Plant Physiology*.

[24] Tchoundjeu Z, Avana ML, Leakey RRB (2002). Vegetative propagation of *Prunus africana*: effects of rooting medium, auxin concentrations and leaf area. *Agroforestry Systems*.

[25] Swamy SL, Puri S, Singh AK (2002). Effect of auxins (IBA and NAA) and season on rooting of juvenile and mature hardwood cuttings of *Robinia pseudoacacia* and *Grewia optiva*. *New Forests*.

[26] Kumlay AM, Eryiğit T (2011). Growth and development regulators in plants: plant hormones. *Igdır University Journal of the Institute of Science and Technology*.

[27] Hepaksoy S (2004). Investigations on micropropagation of some cherry rootstocks I. shoot development and proliferation. *Ege Üniversitesi Ziraat Fakültesi Dergisi*.

[28] Aygün A, Dumanoğlu H (2006). Shoot organogenesis from leaf discs in some quince (*Cydonia oblonga*Mill. *Genotypes, Tarım Bilimleri Dergisi*.

[29] Coşge B, Gürbüz B, Söyler D, Şekeroğlu N (2005). Caper (*Capparis* spp.) cultivation and its importance (A review). *Journal of Crop Research*.

[30] Selby C, Kennedy J, Harvey MR (1992). Adventitious root formation in hypocotyl cuttings of *Picea sitchensis* (Bong.) Carr. the influence of plant growth regulators. *New Phytologist*.

